# Metazoan-like signaling in a unicellular receptor tyrosine kinase

**DOI:** 10.1186/1471-2091-14-4

**Published:** 2013-02-12

**Authors:** Kira P Schultheiss, Barbara P Craddock, Michael Tong, Markus Seeliger, W Todd Miller

**Affiliations:** 1 Department of Physiology and Biophysics, Basic Science Tower, T-6, School of Medicine, Stony Brook University, Stony Brook, NY, 11794-8661, USA; 2 Pharmacology, School of Medicine, Stony Brook University, Stony Brook, NY, 11794-8661, USA

**Keywords:** Tyrosine kinase, Choanoflagellate, Receptor, SH2 domain

## Abstract

**Background:**

Receptor tyrosine kinases (RTKs) are crucial components of signal transduction systems in multicellular animals. Surprisingly, numerous RTKs have been identified in the genomes of unicellular choanoflagellates and other protists. Here, we report the first biochemical study of a unicellular RTK, namely RTKB2 from *Monosiga brevicollis*.

**Results:**

We cloned, expressed, and purified the RTKB2 kinase, and showed that it is enzymatically active. The activity of RTKB2 is controlled by autophosphorylation, as in metazoan RTKs. RTKB2 possesses six copies of a unique domain (designated RM2) in its C-terminal tail. An isolated RM2 domain (or a synthetic peptide derived from the RM2 sequence) served as a substrate for RTKB2 kinase. When phosphorylated, the RM2 domain bound to the Src homology 2 domain of MbSrc1 from *M. brevicollis*. NMR structural studies of the RM2 domain indicated that it is disordered in solution.

**Conclusions:**

Our results are consistent with a model in which RTKB2 activation stimulates receptor autophosphorylation within the RM2 domains. This leads to recruitment of Src-like kinases (and potentially other *M. brevicollis* proteins) and further phosphorylation, which may serve to increase or dampen downstream signals. Thus, crucial features of signal transduction circuitry were established prior to the evolution of metazoans from their unicellular ancestors.

## Background

Metazoan receptor tyrosine kinases (RTKs) respond to a variety of extracellular stimuli, initiating signals that regulate important cellular and developmental processes [1-4]. The phosphotyrosine-based signaling system (consisting of tyrosine kinases, tyrosine phosphatases, and pTyr-binding modules) evolved relatively recently [5-7]; pTyr signaling was originally thought to be unique to metazoans. Remarkably, recent genomic analyses have demonstrated that several unicellular organisms possess numbers of receptor and nonreceptor tyrosine kinases that are comparable to higher metazoans. Choanoflagellates are free-living aquatic protists that represent the closest unicellular relatives to metazoans [8,9]. Tyrosine kinases are abundant in the choanoflagellates *Monosiga brevicollis*, *Monosiga ovata,* and *Salpingoeca rosetta*[10-13]. Tyrosine kinases are also found in even more ancient opisthokonts, including the filasterean *Capsaspora owczarzaki*[14]. Although the physiological functions of these unicellular tyrosine kinases are not yet known, they are presumably involved in the responses to extracellular signals such as the presence of nutrients, ions, or chemical messengers.

The genome of *Monosiga brevicollis* is estimated to encode as many as 128 tyrosine kinases and approximately 380 total protein kinases [10]. (For comparison, the human kinome contains 518 protein kinases, of which 90 are tyrosine kinases). Most of the *Monosiga brevicollis* tyrosine kinases have no identifiable metazoan homologs (the exceptions are nonreceptor tyrosine kinases of the Src, Csk, Abl, and Tec families). As in metazoans, *Monosiga brevicollis* tyrosine kinases never appear as isolated catalytic domains; instead, each tyrosine kinase possesses a repertoire of associated signaling domains. Many of the domain combinations are distinct from any observed in metazoans. There are predicted to be 88 RTKs in *M. brevicollis*; 73 of the RTKs cluster into 15 families (designated RTKA, RTKB, and so on) [10]. While these RTKs possess many of the same features as metazoan RTKs (putative extracellular ligand-binding modules, conserved catalytic residues, and potential sites of autophosphorylation), their overall sequences show limited homology to the families of metazoan RTKs. It is not yet known whether the *M. brevicollis* RTKs (or any unicellular RTKs) are enzymatically active as tyrosine kinases.

**Figure 1:**
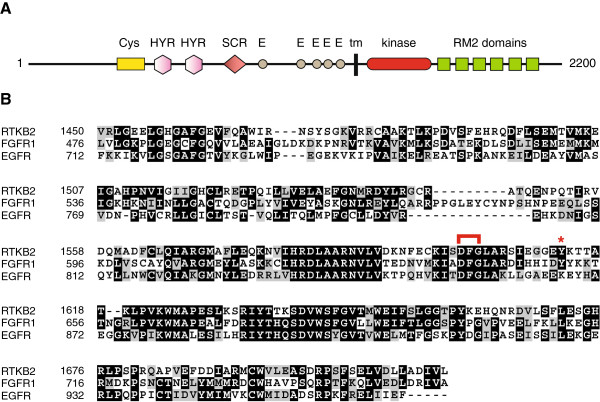
**(A) Domain organization of the *M. brevicollis *RTKB2 kinase **[10]**.** List of domain abbreviations: Cys, cysteine-rich region; HYR, repeats similar to hyalin domains; SCR, short consensus repeat/complement control protein domain; E, epidermal growth factor repeat; tm, transmembrane sequence; RM2, unique *M. brevicollis* domain. **(B)** Amino acid sequence of the RTKB2 kinase domain, aligned with human fibroblast growth factor receptor 1 (FGFR1) and epidermal growth factor receptor (EGFR). Sequences were aligned using the ClustalW program [15] and formatted with BOXSHADE (version 3.21, written by K. Hofmann and M. Baron). The position of the kinase-conserved DFG motif at the beginning of the activation loop is indicated with a red bracket, and an asterisk indicates the position of the potential autophosphorylation site.

The RTKB family of tyrosine kinases from *M. brevicollis* consists of nine members. While the sequences of the RTKB kinase catalytic domains are related, the extracellular regions are quite diverse [10]. Three of the nine RTKB kinases appear to lack important catalytic residues and may function as pseudokinases or scaffolding proteins. Of the six remaining RTKB kinases, four possess a unique modular domain (designated RM2) in their cytoplasmic tails (Figure 1). The RM2 domains contain potential Src-family kinase phosphorylation sites and SH2-binding sites, suggesting that they may link RTK activation with downstream cytoplasmic signals [10]. In this paper, we characterized *Monosiga brevicollis* RTKB2, a kinase with six RM2 domains in its C-tail. We cloned, expressed, and purified the RTKB2 kinase, and conducted biochemical studies of its activity. These studies are the first of an RTK from a unicellular organism.

## Results

*Monosiga brevicollis* RTKB2 is predicted to be a type I transmembrane protein, with a single membrane-spanning region [10] (Figure 1A). The extracellular domain organization of RTKB2 has several features in common with families of metazoan RTKs, although the exact combination of domains has not been observed in any metazoan family. RTKB2 contains a Cys-rich domain similar to those seen in the TNF receptor and to the furin-like domains of EGFR and insulin receptor [4]. Two divergent repeats similar to hyalin are present; these modules are structurally related to the immunoglobulin-like fold seen in many metazoan RTKs [16]. RTKB2 contains a short consensus repeat (SCR)/complement control protein domain, as seen in a variety of complement and adhesion proteins. Towards the C-terminus of the extracellular domain, RTKB2 contains five EGF-like modules, as seen in (for example) the Tie and Eph families of metazoan RTKs. As is true for all *Monosiga brevicollis* RTKs, the extracellular ligand is unknown.

The predicted intracellular portion of RTKB2 contains a single tyrosine kinase catalytic domain. The kinase domain of RTKB2 has limited homology to known families of metazoan RTKs, but possesses the catalytically important residues found in all tyrosine kinases (Figure 1B). RTKB2 has a single tyrosine residue in the predicted activation loop, C-terminal to the kinase-conserved DFG motif (Figure 1B). The C-terminus of RTKB2 contains six copies of a unique domain designated RM2. RM2 domains are composed of approximately 80 amino acid residues. They are found in the cytoplasmic tails of four of the nine *Monosiga brevicollis* RTKB tyrosine kinases, but they are not present in any other sequence in the protein database, whether from *Monosiga*, metazoans, or any other organism [10]. RM2 domains contain tyrosine residues that are predicted to serve as phosphorylation sites as well as SH2-binding sites (described in more detail below).

**Figure 2:**
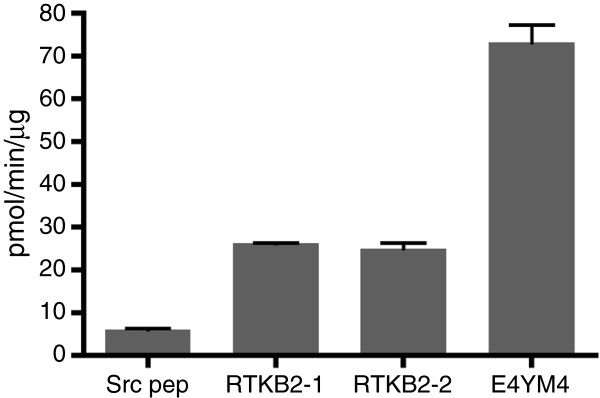
**Peptide phosphorylation by purified RTKB2 kinase.** The enzymatic activity of RTKB2 (1 μM) towards four synthetic peptides (750 μM) was measured using the phosphocellulose paper binding assay. Reactions were carried out for 15 minutes at 30°C. Error bars indicate standard deviations.

To test whether the predicted RTKB2 protein is an active tyrosine kinase, we used PCR with kinase-specific primers to amplify the cDNA encoding the catalytic domain (residues 1450–1724) from a *Monosiga brevicollis* cDNA library. We cloned the RTKB2 kinase sequence into a baculovirus expression vector, infected *Spodoptera frugiperda* (Sf9) insect cells with the recombinant baculovirus, and purified the protein (Additional file 1: Figure S1). Using the phosphocellulose paper binding assay, we tested RTKB2 kinase activity towards several synthetic peptides (Figure 2). RTKB2 was highly active, with a specific activity similar to other purified RTK kinase domains (e.g., the kinase domain of insulin-like growth factor I receptor [17]). The highest activity was observed with a peptide (E4YM4) containing the EEEEYMMMM motif that was selected from a synthetic peptide library as a highly efficient insulin receptor substrate [18]. This activity (73 pmol/min/μg) is comparable to the activity of *Monosiga brevicollis* MbSrc1 toward its preferred substrates (145 pmol/min/μg; ref. *10*). We also tested two peptides with sequences corresponding to predicted phosphorylation sites within RTKB2 RM2 domains. RTKB2-1 is derived from the fifth RM2 domain, while RTKB2-2 is derived from the sixth RM2. Both peptides were phosphorylated by RTKB2 kinase, consistent with the possibility that these sites on the RTKB2 C-tail serve as autophosphorylation sites. We previously showed that these peptides are also phosphorylated by the *Monosiga* Src-like kinase MbSrc1 [10].

**Figure 3:**
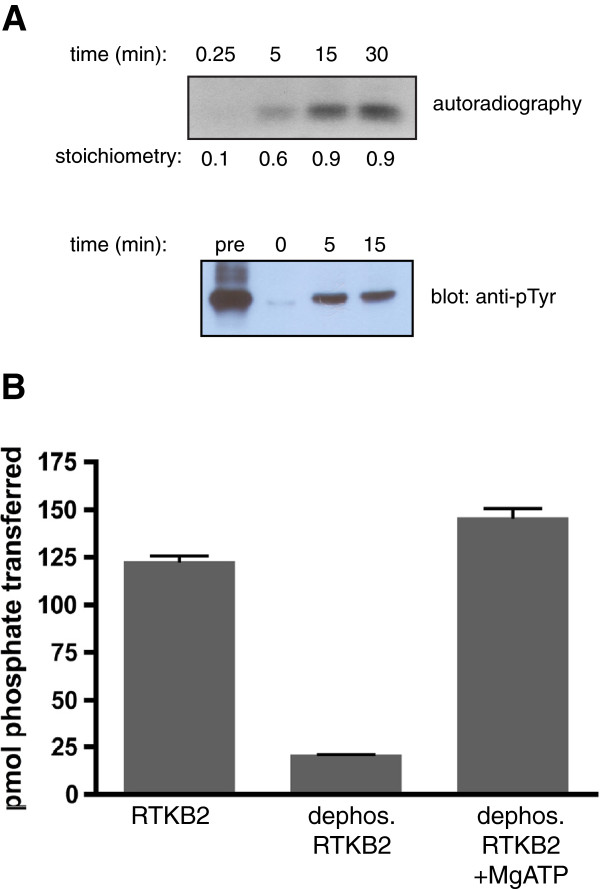
**RTKB2 autophosphorylation. (A)** RTKB2 kinase was first treated with GST-YOP phosphatase, then incubated for the indicated times in the presence of 0.5 mM ATP. Top panel: the autophosphorylation reaction contained [γ-^32^P]-ATP, and the reaction was analyzed by SDS-PAGE and autoradiography. Incorporation of ^32^P into RTKB2 kinase was also measured by scintillation counting, and the stoichiometry (mol phosphate/mol RTKB2) is presented below the gel. Bottom panel: unlabeled ATP was used in the reaction, which was analyzed by SDS-PAGE and Western blotting with anti-pTyr antibody. Also analyzed were samples of RTKB2 kinase before YOP treatment (“pre”) and after YOP treatment but before autophosphorylation (0 min.). **(B)** The activity of Sf9-purified RTKB2 towards the E4YM4 synthetic peptide was measured either directly (RTKB2), after treatment with YOP tyrosine phosphatase (dephos. RTKB2), or after treatment with YOP followed by an autophosphorylation reaction (30 min at 30°C; dephos. RTKB2 + MgATP). Activity was measured using the phosphocellulose paper binding assay.

Many RTKs are regulated by autophosphorylation at one or more tyrosines within the activation loop, a flexible segment between the two lobes of the kinase catalytic domain [2]. RTKB2 has a single tyrosine in the predicted activation loop (Figure 1B). To test for RTKB2 autophosphorylation, we first treated the purified enzyme with *Yersinia* tyrosine-specific YOP phosphatase. Next, we incubated the dephosphorylated RTKB2 with magnesium and [γ-^32^P]-ATP and followed the reaction by SDS-PAGE and autoradiography (Figrue 3A, top panel). Labeled phosphate was incorporated into RTKB2 after 5–15 minutes under these conditions. The stoichiometry of phosphorylation after 15 minutes was 0.93 mol phosphate/mol protein. In a parallel experiment, the time-course of autophosphorylation was followed by Western blotting with anti-phosphotyrosine antibody (Figure 3A, bottom panel). In this experiment, the signal for pre-YOP treated RTKB2 was stronger than the signal for autophosphorylated RTKB2, raising the possibility that multiple sites are phosphorylated in Sf9 cells. To determine whether autophosphorylation influences RTKB2 kinase activity, we carried out a peptide phosphorylation assay with purified RTKB2, YOP-dephosphorylated RTKB2, and enzyme that had been allowed to re-phosphorylate as described above. Dephosphorylated RTKB2 lost significant kinase activity as compared to the starting sample, but the activity was regained upon re-phosphorylation (Figure 3B). These results are consistent with a role for RTKB2 autophosphorylation in the control of enzymatic function.

**Figure 4:**
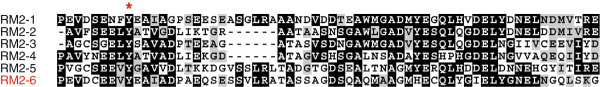
**Alignment of the amino acid sequences of the six RM2 domains **[10]**.** Sequences were aligned using the ClustalW program [15] and formatted with BOXSHADE (version 3.21, written by K. Hofmann and M. Baron). The conserved tyrosine residue is indicated by a red asterisk. The sixth RM2 domain, shown in red, was chosen for further analysis by bacterial expression.

The six RM2 domains in the C-tail of RTKB2 contain tyrosines near their N-termini that are predicted to serve as phosphorylation and SH2-binding sites (Figure 4) [10]. As shown above in Figure 2, synthetic peptides derived from two of the domains are indeed substrates for RTKB2 kinase. To study an intact RM2 domain, we synthesized the gene corresponding to the sixth RM2 domain (the sequence of RM2-6 is presented in Figure 4). The sixth RM2 domain was chosen because it contains a predicted Src phosphorylation/SH2-binding site that conforms well to the consensus (EEVYEAI), and because the RTKB2-2 peptide (Figure 2) corresponds to this site. We cloned the cDNA into a bacterial expression vector, expressed the protein in *E. coli* cells, and purified it by metal affinity chromatography (Additional file 2: Figure S2A). Despite a predicted molecular weight of 10,089 daltons, purified RM2-6 migrated with an apparent molecular weight of approximately 20,000 daltons on SDS-PAGE (Additional file 2: Figure S2A). The aberrant mobility of RM2-6 may be due to the highly acidic amino acid composition (pI = 3.9). We confirmed the molecular weight of the bacterially-expressed RM2 domain by matrix-assisted laser desorption/ionization mass spectrometry (Additional file 2: Figure S2B).

**Figure 5:**
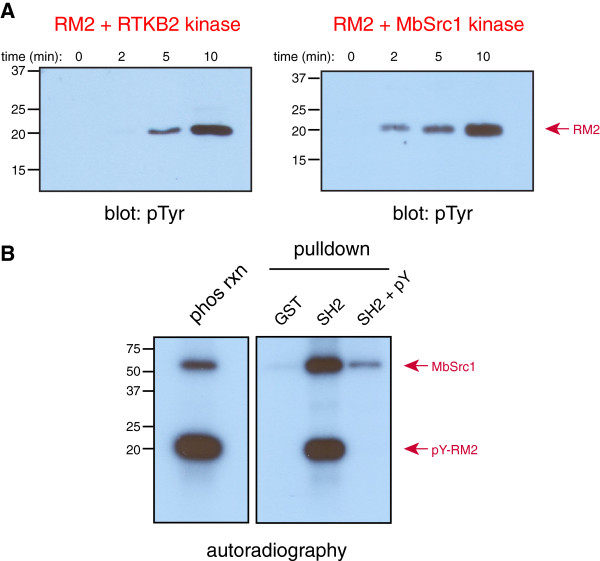
**The RM2 domain acts as a kinase substrate and SH2-binding site. (A)** Left panel: purified RM2-6 (1 μM) was incubated with RTKB2 (250 nM) in kinase assay buffer containing 0.8 mM ATP at 30°C for the indicated times. Aliquots were removed, boiled with SDS-PAGE sample buffer, and analyzed by Western blotting with anti-pTyr antibody. Right panel: similar experiments were carried out with purified MbSrc1 kinase. **(B)** Purified RM2-6 was phosphorylated by MbSrc1 in the presence of [γ-^32^P]-ATP. A sample of the reaction mixture is shown in the left panel. Right panel: the reaction mixture containing phosphorylated RM2 domain was incubated for 30 minutes with 25 μl of immobilized GST or a GST-MbSrc1 SH2 domain fusion protein [20] in binding buffer (50 mM Tris, pH 7.5, 250 mM NaCl, 5 mM EDTA, 0.1% Triton X-100, total volume = 200 μl). In one reaction, an SH2-binding synthetic peptide (Glu-Pro-Gln-pTyr-Glu-Glu-Ile-Pro-Ile-Lys-Gln; labeled “pY” on the gel) was added at a concentration of 100 μM as a competitor. The glutathione-agarose beads were washed 4 times with 1 ml of binding buffer. Proteins were eluted by boiling with SDS-PAGE sample buffer and visualized by autoradiography.

To determine whether the isolated RM2 domain could serve as a substrate for RTKB2 kinase, we incubated the two purified proteins with Mg-ATP and followed the reaction by anti-pTyr Western blotting (Figure 5A, left panel). Tyrosine-phosphorylated RM2-6 was detected after 5–10 minutes in this reaction, consistent with the role of RM2 domains as autophosphorylation sites. The purified RM2 domain could also be phosphorylated by *M. brevicollis* MbSrc1 kinase (Figure 5A, right panel). We determined the stoichiometry of RM2-6 phosphorylation by MbSrc1 in a parallel reaction with [γ-^32^P]-ATP. After 5 minutes, the stoichiometry was 2.02 ± 0.13 moles of phosphate per mole of RM2-6 domain. RM2-6 has three potential tyrosine phosphorylation sites; these results raise the possibility of multisite phosphorylation in the six RM2 domains in the C-terminus of RTKB2. The Tyr-Glu-Ala-Ile sequence within RM2-6 fits the consensus sequence for binding to the SH2 domains of Src-family kinases [19]. The MbSrc1 SH2 domain has a similar binding preference as its metazoan counterparts [20]. To test for MbSrc1 SH2 binding, we first phosphorylated RM2-6 using RTKB2 and [γ-^32^P]-ATP. Next, we incubated the pY-RM2 with an immobilized protein consisting of glutathione S-transferase (GST) fused to the SH2 domain of MbSrc1 (Figure 5B). The phosphorylated RM2 domain bound to the MbSrc1 SH2 domain, as detected by autoradiography. When the binding reaction was carried out in the presence of a high-affinity SH2 binding peptide (pYEEI), binding between RM-6 and the MbSrc1 SH2 domains was drastically reduced (Figure 5B). These results are consistent with a model in which RTKB2 autophosphorylation initiates the formation of a signaling complex, potentially containing MbSrc1 (by analogy to metazoan RTK signaling) and additional SH2-containing *Monosiga* proteins.

**Figure 6:**
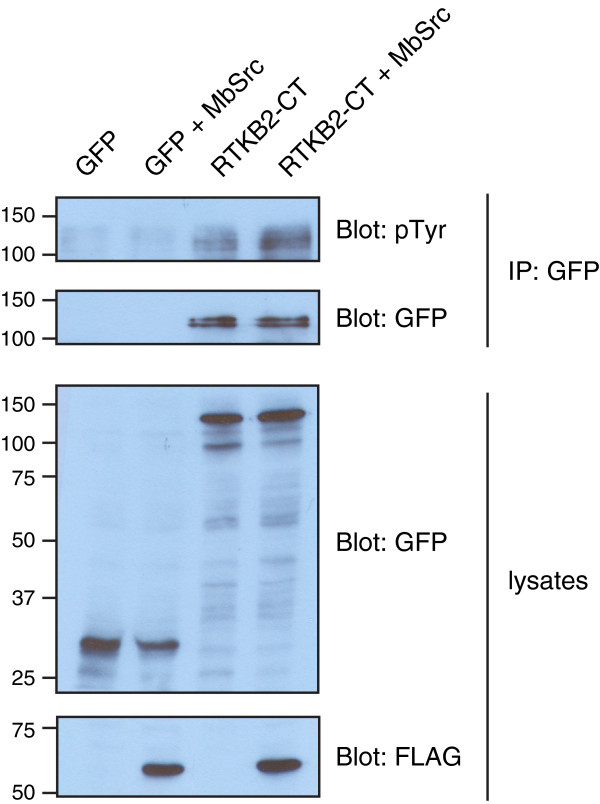
**Expression of the C-terminus of RTKB2 in mammalian cells.** SYF cells were transiently transfected with plasmids encoding GFP or GFP-RTKB2-C-terminus (alone or together with a plasmid encoding FLAG-tagged MbSrc1). Top panels: anti-GFP immunoprecipitates were separated by SDS-PAGE and analyzed by Western blotting with anti-phosphotyrosine antibodies. The membrane was subsequently stripped and reprobed with anti-GFP antibodies. The bottom two panels show expression of GFP, GFP-RTKB2-CT, and FLAG-MbSrc1 in the SYF cell lysates.

To study RM2 domain phosphorylation in a cellular context, we amplified a cDNA encoding the C-terminal tail of the RTK (all six RM2 domains), and cloned it into a mammalian expression vector to produce a fusion with green fluorescent protein (GFP). We expressed the protein in Src/Yes/Fyn triple knockout fibroblast cells (SYF cells), which lack all Src family kinases [21]. Fluorescence microscopy showed that the GFP-RTKB2-CT has a diffuse cytoplasmic localization (Additional file 3: Figure S3). Next, we expressed GFP-RTKB2-CT in SYF cells alone or in the presence of Flag-tagged MbSrc1 kinase (Figure 6). We isolated RTKB2 by anti-GFP immunoprecipitation and analyzed tyrosine phosphorylation by Western blotting. RTKB2-CT was weakly tyrosine-phosphorylated in these experiments, and the addition of MbSrc1 gave only a modest increase in phosphorylation (Figure 6). We were unable to detect a complex between RTKB2-CT and MbSrc1 by co-immunoprecipitation (data not shown). While these experiments confirm the ability of the RTKB2 C-tail to be phosphorylated, it is likely that ligand-stimulated RTKB2 kinase would produce much higher levels of tyrosine phosphorylation. Furthermore, the lack of membrane-anchoring motifs in the RTKB2-CT and MbSrc1 constructs likely reduces the efficiency of their interaction.

The unique sequences of the RM2 domains suggest that they may serve as novel signaling modules. Many eukaryotic signaling domains adopt defined structures, even when removed from their parental proteins [22]. In order to evaluate the solution structure of ^15^N labeled RM2, a ^1^H-^15^N HSQC spectrum was acquired (Additional file 4: Figure S4). The spectrum of RM2 illustrates that the chemical shift of the backbone amide resonances display narrow dispersion, cluster and overlap around the center of the spectrum, while the line shapes are sharp. These features are typical of a disordered protein. Furthermore, the number of backbone amide resonances is lower than the expected count of 103 (excluding the amino terminal residue and prolines) for His10 tagged RM2, presumably because the resonances are extensively overlapped due to the disordered state of the RM2 domain. We attempted to model the structure of the RM2-6 domain using the web-based Protein Homology/Analogy Recognition Engine (Phyre) (http://www.sbg.bio.ic.ac.uk/phyre2). Phyre predicted that 52% of the RM2-6 domain is disordered, and secondary structural elements were predicted with low confidence. Collectively, our data suggest that the RM2 domains of RTKB2 serve as kinase/SH2 binding sites, and that pTyr-SH2 binding is the main determinant for interaction, rather than a well-defined protein-protein interface.

## Discussion

The abundance and diversity of receptor and nonreceptor tyrosine kinases in the unicellular choanoflagellate *Monosiga brevicollis* rival that of any metazoan [10,11,23]. The 88 RTKs found in the genome of *M. brevicollis* possess a wide variety of domain organizations. The divergent architectures of the choanoflagellate RTKs were likely generated by gene duplication and domain shuffling [24,25]. The *M. brevicollis* RTKs have no direct homologs in multicellular organisms. In contrast, sponges, which are regarded as the oldest surviving metazoan lineage, possess most of the RTK families found in higher metazoans [26-28]. The genome of the sponge *Amphimedon queenslandica* contains 150 RTK genes, including kinase domains from six known animal families: epidermal growth factor receptor (EGFR), Met, discoidin domain receptor (DDR), ROR, Eph, and Sevenless [26]. The sponge *Oscarella carmela* possesses a similar array of homologs, and RTKs with homology to the receptors for insulin-like growth factor I and fibroblast growth factor [28]. This is consistent with a model in which the common ancestor between choanoflagellates and metazoans had RTKs, but the animal cell-specific families of RTKs developed after the split between the two groups [10].

RTKB2, the tyrosine kinase studied here, is one of the nine RTKB-family kinases from *M. brevicollis*. As the most primitive RTK to be yet studied, these results shed light on the evolution of biochemical function in the receptor tyrosine kinase superfamily. RTKB2 is active as a tyrosine kinase, and the intrinsic enzymatic function of the RTKB2 catalytic domain is high towards synthetic peptides, particularly the IR/IGF1R family peptide substrate E4YM4 (Figure 2). RTKB2 also catalyzes autophosphorylation, an event that increases the activity of the enzyme (Figure 3). RTKB2 possesses a single tyrosine residue within the predicted activation loop (Figure 1B). Activation loops are one of the distinguishing features of eukaryotic protein kinases [29]. They are flexible, dynamic segments that are often stabilized in the active conformation by addition of one or more phosphates (either through autophosphorylation or through the action of another kinase). The control of RTKB2 activity by autophosphorylation indicates that this mode of regulation was present in primitive RTKs before the evolution of multicellular animals over 600 million years ago.

The C-terminal tail of RTKB2 contains 6 copies of the RM2 domain, a unique region of ≈ 80 residues that has not been found outside of the RTKB family in *M. brevicollis* (Figure 4). RTKB1, RTKB3, and RTKB4 each have one copy of the RM2 domain C-terminal to their tyrosine kinase domains [10]. Each of the RM2 domains has a tyrosine residue preceded by one or more negatively-charged amino acids. Scansite prediction indicated that these could serve as Src phosphorylation sites and/or SH2-binding sites [10]. Our data show that an isolated RM2 domain is phosphorylated by the RTKB2 kinase or by MbSrc1, a Src-family nonreceptor tyrosine kinase from *M. brevicollis* (Figure 5). Small synthetic peptides derived from the potential phosphorylation sites from two of the RTKB2 RM2 domains are also substrates (Figure 2). The tyrosine phosphorylated RM2 domain binds specifically to the SH2 domain of MbSrc1 (Figure 5B), suggesting that phosphorylation of one or more RM2 domains can recruit cytoplasmic tyrosine kinases and other cellular proteins to propagate the RTKB2 signal. In contrast to other modular signaling domains (e.g., SH2 or SH3), the isolated RM2 domain appears to lack an ordered structure when it is removed from the context of the surrounding protein (Additional file 4: Figure S4). These binding sites at the C-terminus of RTKB2 may serve a similar recruitment function as the tyrosine motifs found in the short, unstructured cytoplasmic tails of the thrombopoietin or erythropoietin receptors [30,31] or the phosphorylation sites in the C-termini of RTKs such as the epidermal growth factor receptor.

Binding of the *M. brevicollis* MbSrc1 kinase to the phosphorylated RM2 domain raises the possibility that receptor and nonreceptor tyrosine kinase signaling are linked, as in metazoans. A classic example of the linkage in mammalian cells is observed in the role for Src in relaying the signal through the platelet-derived growth factor (PDGF) family of receptors. Src stably associates with the cytoplasmic portion of the PDGF receptor, leading to enhanced Src activity [32-34]. For PDGFRa, Src is required for the phosphorylation of the adaptor protein Shc [32]. After binding to PDGFRb, Src phosphorylates a tyrosine residue on the receptor; this inhibits a signaling pathway leading to motility, but increases mitogenic signaling [33]. MbSrc1 phosphorylates the C-tail of RTKB2 weakly in the absence of an activating signal (Figure 6). Our results are consistent with a model in which RTKB2 activation (by an unknown signal) stimulates receptor autophosphorylation within the RM2 domains. This leads to MbSrc1 recruitment and further phosphorylation, which may serve to increase or dampen specific downstream signals. Identifying the nature of these signals will require the development of methodology to manipulate gene function in choanoflagellates.

## Conclusions

We conducted the first biochemical study of a unicellular receptor tyrosine kinase. We cloned, expressed, and purified the RTKB2 kinase, and showed that it is enzymatically active. The activity of RTKB2 is regulated by autophosphorylation. The receptor possesses six copies of a unique domain (designated RM2) in its C-terminal tail. An isolated RM2 domain was a substrate for RTKB2 kinase, and the phosphorylated RM2 domain bound to the SH2 domain of a Src family kinase. Thus, this unicellular signaling system contains many of the features found in metazoan RTK pathways.

## Methods

### cDNA cloning

The protein sequence of RTKB2 was predicted from release 1.0 of the *Monosiga brevicollis* genome (http://genome.jgi-psf.org/Monbr1/Monbr1.home.html) [23]. The kinase domain (residues 1450–1724) or C-terminal tail (residues 1722–2200) were amplified by PCR from an *M. brevicollis* cDNA library [35]. For baculovirus expression, the kinase cDNA was cloned into the BamHI and XbaI sites of pFastbac-Htb (Invitrogen). For mammalian cell expression, the C-terminal tail cDNA was cloned into the EcoRI and BamHI sites of plasmid pEGFP-C1 (Clontech). For bacterial expression of RM2-6, the gene was synthesized with a sequence optimized for *E. coli* (GenScript) and cloned into plasmid pET-15b.

### Protein expression and purification

His-tagged RTKB2 kinase was produced in *Spodoptera frugiperda* (Sf9) insect cells using the Bac-to-Bac system (Invitrogen), using methods developed for other tyrosine kinases [17,20]. The enzyme was purified by column chromatography with nickel-nitrilotriacetic acid (NiNTA) resin (Qiagen) and stored in 40% glycerol at −20°C. His-tagged RM2-6 was expressed in 1-liter *E. coli* cultures and purified by Ni-NTA chromatography.

The following conditions were used to produce labeled RM2-6 for NMR: the RM2-6 domain cDNA was cloned into pSKB3-His10 (customized pET28 vector) as a fusion featuring an N-terminal His10 tag and TEV protease cleavage site. For ^15^N labeled RM2-6 expression, pSKB3-His10-RM2 was transformed into *E.coli* BL21 (DE3) and selected for with Kanamycin (50 μg/μL), subsequently 2L of ^15^N enriched M9 minimal media containing ^15^NH_4_Cl as the sole source of nitrogen was inoculated with the cells and grown at 37°C. At O.D_600nm ~_ 0.4, the cultures were cooled to 16°C and induced with 0.5 mM IPTG at O.D_600nm__~_ 0.7-0.8. Expression was allowed to proceed overnight.

Cells were harvested by centrifugation at 3000 g for 10 minutes at 4°C, resuspended in lysis buffer (20 mM Tris pH 8, 500 mM NaCl, 5% glycerol), lysed by sonication on ice, centrifuged for 30 min at 18000g and then purified by Ni^2+^ NTA affinity chromatography. RM2-6 was eluted using a linear 0–100% imidazole gradient (lysis buffer plus 500 mM imidazole). Fractions containing RM2-6 were pooled and diluted 2-fold with 20 mM Tris pH 8, then purified further by anion exchange on a Q column. RM2-6 was eluted over a linear 0–40% NaCl gradient (QA buffer: 20 mM Tris pH 8, 5% glycerol, 1 mM DTT, QB buffer: same as QA buffer plus 1 M NaCl). Subsequent RM2-6 containing fractions were pooled and buffer exchanged into 50 mM NaH_2_PO_4_/Na_2_HPO_4_ pH 7, 150 mM NaCl, 1 mM DTT, then concentrated to 800 μM. A 500 μL RM2-6 sample at 720 μM was prepared with 10% ^2^H_2_O for NMR analysis.

### Tyrosine kinase assays

RTKB2 synthetic peptide assays were performed with [γ-^32^P]-ATP using the phosphocellulose paper binding assay [36,37]. Reaction mixtures contained 20 mM Tris–HCl (pH 7.4), 10 mM MgCl_2_, 0.1 mM Na_2_VO_4_, 0.5 mM DTT, 0.25 mM ATP, varying concentrations of peptide substrate, and [γ-^32^P]-ATP (200–400 cpm/pmol). The sequences of the peptides tested were: Src peptide, AEEEIYGEFEAKKKKG ; RTKB2-1, SEEVYGAVVDKKK; RTKB2-2, AEEVYEAIADKKK; insulin receptor substrate (E4YM4), KKEEEEYMMMMG. RTKB2 autophosphorylation was measured after treatment with a glutathione S-transferase (GST) fusion protein containing *Yersinia* YOP phosphatase. Reaction mixtures contained 10 mM MgCl_2_ and 0.5 mM unlabeled ATP (for Western blotting experiments) or 0.5 mM [γ-^32^P]-ATP (for analysis by autoradiography).

### NMR experiments

A ^1^H-^15^N HSQC spectrum of ^15^N labeled RM2-6 was acquired on a Bruker 700 MHz spectrometer at 25°C using 16 scans, data points TD2 = 2048, TD1 = 128, with spectral widths of 11160.71 Hz (^1^H) and 2483.23 Hz (^15^N) respectively. The spectrum was processed with Topspin and the spectrum figure was created using CCPNMR analysis 2.1.

### Cell transfection and western blotting

SYF cells were cultured in Dulbecco’s modified Eagle’s medium plus 10% fetal bovine serum at 37°C in 5% CO2. Cells were transfected using TransIT polyamine transfection reagent (Mirus) according to the manufacturer’s instructions. Cells were lysed in buffer containing 10 mM Tris–HCl, pH 7.4, 50 mM NaCl, 5 mM EDTA, 1% TritonX-100, 50 mM NaF, 2 mM Na_3_VO_4_, 1mMPMSF, 1mg/ml aprotinin, and 1mg/ml leupeptin. After centrifugation, protein concentrations were determined using a Bio-Rad protein assay. For immunoprecipitation experiments, lysates (1 mg total protein) were precleared by mixing with 50 ml of protein A-agarose in lysis buffer for one hour at 4°C. After pre-clearing, 2 μg of anti-GFP antibody was added to the lysate and incubated for one hour at 4°C with rocking. Antibody protein complexes were collected with 50 μl protein A beads. The beads were washed 5 times in lysis buffer and boiled in 40 μl gel loading buffer. After separation by SDS-PAGE, proteins were transferred to PVDF membrane and analyzed by Western blotting. Western blotting experiments were carried out with the following antibodies: anti-phosphotyrosine antibody (4G10, Upstate), anti-Flag antibody (M2, Sigma) and anti-GFP antibody (Santa Cruz). Detection was by enhanced chemiluminescence (GE Healthcare).

For immunofluorescence miscroscopy, cells expressing GFP-RTKB2 were grown on 35mm glass bottom dishes (In Vitro Scientific). The cells were first washed with 1x PBS, then fixed with dilute 3.7% formaldehyde in 1x PBS for 15 minutes at room temperature. The fixed cells were washed several times with 1x PBS then mounted on coverslips using VECTASHIELD medium (Vector Laboratories). The GFP expressing cells were visualized by epifluorescence microscopy using a Zeiss Axiovert 200M inverted microscope and a Plan Apochromat 63x/1.40 oil objective. Images were captured using a GFP-Chroma Filter Set, AxioVision software and an AxioCam MRm CCD camera.

## Availability of supporting data

Data supporting the results of this article are included within the article and in Additional file 1: Figure S1, Additional file 2: Figure S2, Additional file 3: Figure S3 and Additional file 4: Figure S4.

## Abbreviations

SH2: Src homology 2; DTT: Dithiothreitol; EDTA: Ethylenediamine tetraacetic acid; EGF: Epidermal growth factor; GFP: Green fluorescent protein; GST: Glutathione S-tranferase; HSQC: Heteronuclear single quantum coherence; IPTG: Isopropyl-b-D-1-thiogalactopyranoside; NiNTA: Nickel-nitrilotriacetic acid; PCR: Polymerase chain reaction; PDGF: Platelet derived growth factor; PMSF: Phenylmethylsulfonyl fluoride; PVDF: Polyvinylidene fluoride; RTK: Receptor tyrosine kinase; SYF: Src/Yes/Fyn deficient cells; TEV: Tobacco etch virus protease.

## Competing interests

The authors declare that they have no competing interests.

## Authors’ contributions

KPS, BPC, and WTM carried out enzymology studies, KPS and WTM carried out cellular studies, and MT and MS performed NMR and modeling studies. WTM designed the study and drafted the manuscript. All authors read and approved the final manuscript.

## Supplementary Material

Additional file 1: Figure S1 SDS-PAGE analysis of RTKB2 kinase domain. Lanes 1, 2, and 3 show 0.3, 1.5, and 4.0 μg of purified RTKB2 kinase. Detection: Coomassie blue staining. Click here for file

Additional file 2: Figure S2 (A) SDS-PAGE of purified RM2-6 (Coomassie staining). (B) MALDI-MS analysis of RM2-6 domain. Click here for file

Additional file 3: Figure S3 Immunofluorescence microscopy of SYF cells expressing GFP (top) or GFP-RTKB2-cyto (bottom). Click here for file

Additional file 4: Figure S4 ^1^H ^15^N HSQC spectrum of the RM2-6 domain from Monosiga brevicollis RTKB2 kinase. The spectrum indicates that RM2-6 is disordered because its backbone amide resonances exhibit a narrow and clustered chemical shift environment that is typical of an unfolded protein. Click here for file
