# Supplementary figures and images for: Metazoan-like signaling in a unicellular receptor tyrosine kinase
Source: BMC Biochem. 2013 Feb 12;14:4. doi: 10.1186/1471-2091-14-4 (PMC3584944; doi:10.1186/1471-2091-14-4)

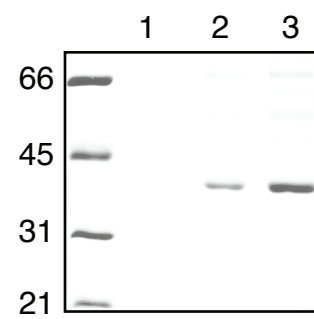

Figure S1

Supplement: Additional file 1: Figure S1 — SDS-PAGE analysis of RTKB2 kinase domain. Lanes 1, 2, and 3 show 0.3, 1.5, and 4.0 μg of purified RTKB2 kinase. Detection: Coomassie blue staining. [file 1471-2091-14-4-S1.pdf]

**A**

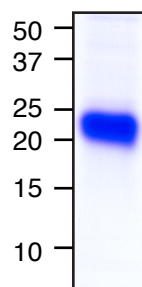

**B**

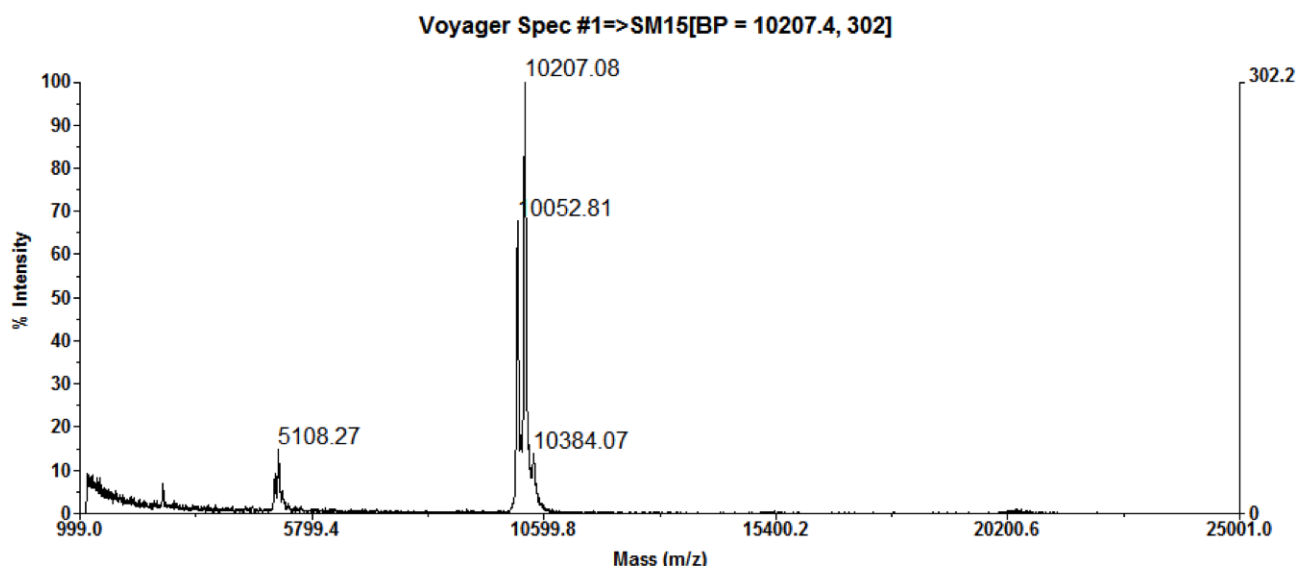

Figure S2

Supplement: Additional file 2: Figure S2 — (A) SDS-PAGE of purified RM2-6 (Coomassie staining). (B) MALDI-MS analysis of RM2-6 domain. [file 1471-2091-14-4-S2.pdf]

**A**

GFP

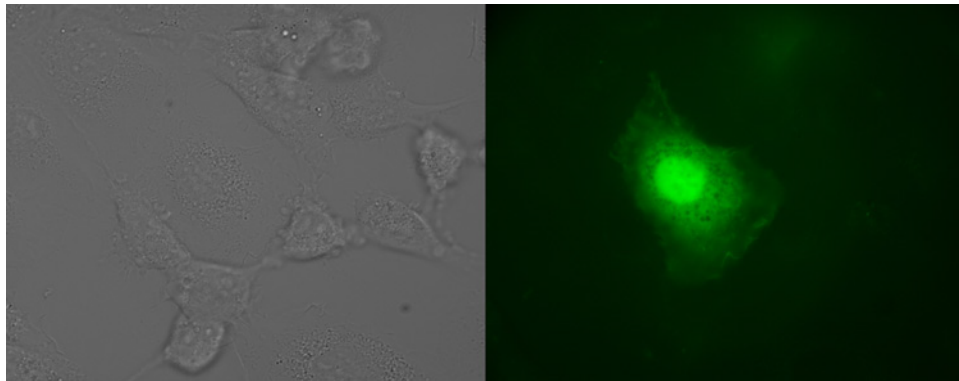

**B**

GFP-RTKB2-cyto

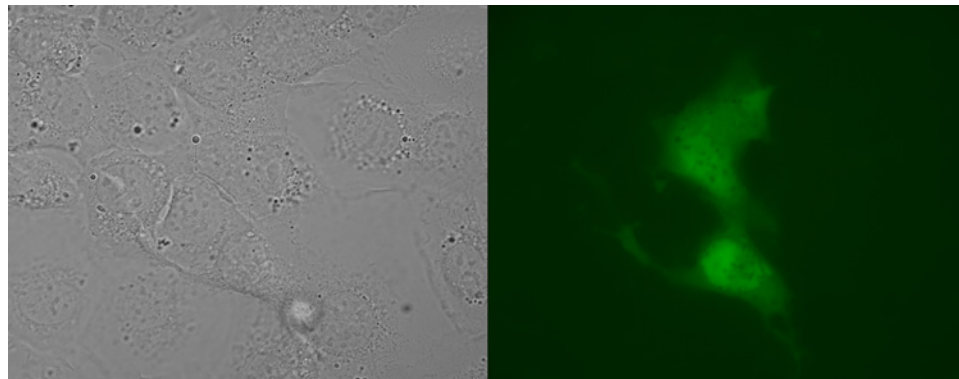

Figure S3

Supplement: Additional file 3: Figure S3 — Immunofluorescence microscopy of SYF cells expressing GFP (top) or GFP-RTKB2-cyto (bottom). [file 1471-2091-14-4-S3.pdf]

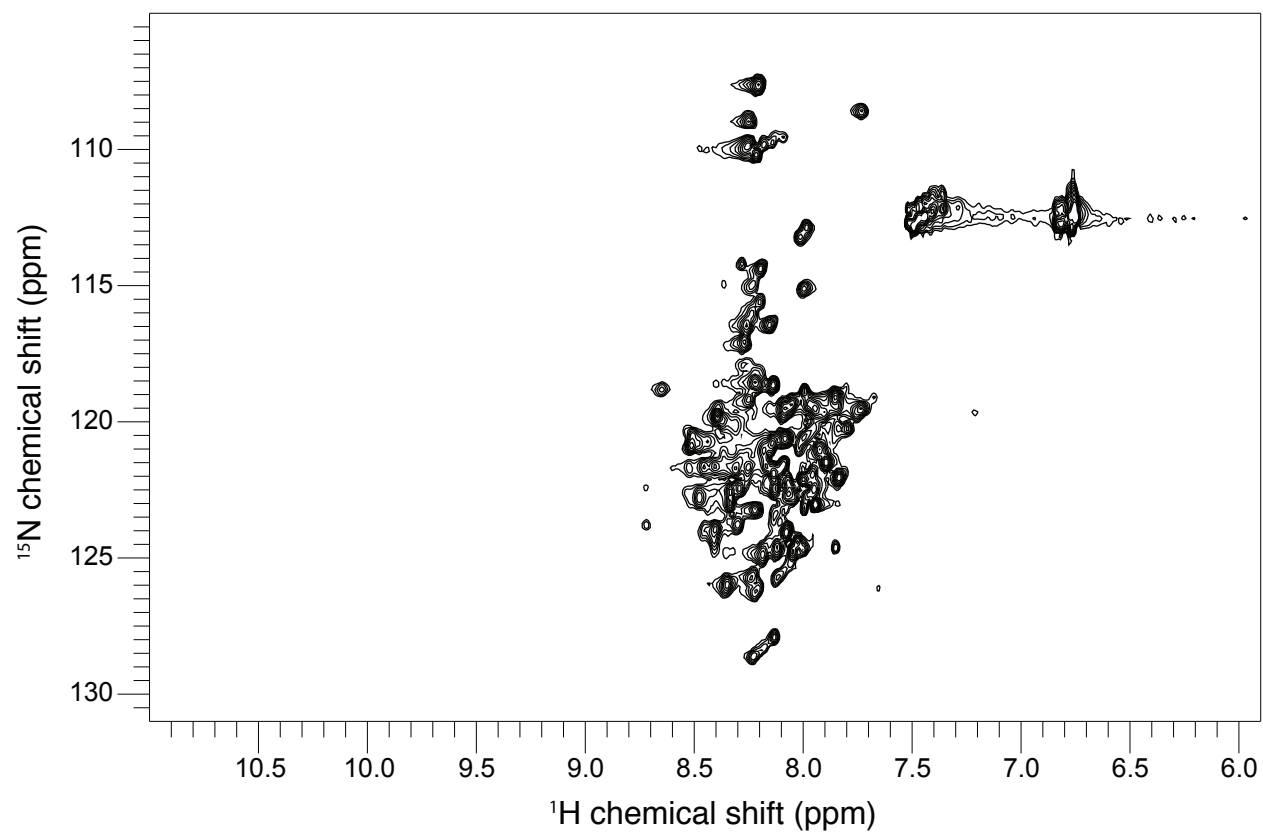

Figure S4

Supplement: Additional file 4: Figure S4 — 1H 15N HSQC spectrum of the RM2-6 domain from Monosiga brevicollis RTKB2 kinase. The spectrum indicates that RM2-6 is disordered because its backbone amide resonances exhibit a narrow and clustered chemical shift environment that is typical of an unfolded protein. [file 1471-2091-14-4-S4.pdf]
